# Combination of Human Leukocyte Antigen and Killer Cell Immunoglobulin-Like Receptor Genetic Background Influences the Onset Age of Hepatocellular Carcinoma in Male Patients with Hepatitis B Virus Infection

**DOI:** 10.1155/2013/874514

**Published:** 2013-11-10

**Authors:** Ning Pan, Jie Qiu, Hang Sun, Fengqin Miao, Qian Shi, Jinhuan Xu, Wei Jiang, Hui Jin, Wei Xie, Youji He, Jianqiong Zhang

**Affiliations:** ^1^Department of Pathogenic Biology and Immunology, Southeast University Medical School, 87 Dingjiaqiao Road, Nanjing, Jiangsu 210009, China; ^2^Key Laboratory of Developmental Genes and Human Disease, Ministry of Education, Southeast University, Nanjing, Jiangsu 210009, China; ^3^The Second Affiliated Hospital of Southeast University, Nanjing, Jiangsu 210000, China; ^4^Department of Epidemiology, School of Public Health, Southeast University, Nanjing, Jiangsu 210009, China

## Abstract

To investigate whether killer cell immunoglobulin-like receptor *(KIR)* and human leukocyte antigen *(HLA)* genetic background could influence the onset age of hepatocellular carcinoma (HCC) in patients with hepatitis B virus (HBV) infection, one hundred and seventy-one males with HBV-related HCC were enrolled. The presence of 12 loci of *KIR* was detected individually. *HLA-A, -B, and -C* loci were genotyped with high resolution by a routine sequence-based typing method. The effect of each *KIR* locus, *HLA* ligand, and *HLA-KIR* combination was examined individually by Kaplan-Meier (KM) analysis. Multivariate Cox hazard regression model was also applied. We identified *C1C1-KIR2DS2/2DL2* as an independent risk factor for earlier onset age of HCC (median onset age was 44 for *C1C1-KIR2DS2/2DL2* positive patients compared to 50 for negative patients, *P* = 0.04 for KM analysis; HR = 1.70, *P* = 0.004 for multivariate Cox model). We conclude that *KIR* and *HLA* genetic background can influence the onset age of HCC in male patients with HBV infection. This study may be useful to improve the current HCC surveillance program in HBV-infected patients. Our findings also suggest an important role of natural killer cells (or other *KIR*-expressing cells) in the progress of HBV-related HCC development.

## 1. Introduction

Primary liver cancer, particularly hepatocellular carcinoma (HCC), is the fifth most common cancer worldwide and the third most common cause of cancer mortality. The curative therapy for HCC is surgical resection, for which only the patients with early stage tumor are eligible. However, most cases of HCC patients are detected in their late stages and become ineligible for surgical resection. Globally, more than 50% of HCC are due to persistent hepatitis B virus (HBV) infection. In the hyperendemic areas of HCC, such as China and Africa, chronic HBV infection contributes to at least 80% of HCC cases [1]. Periodic screening for HCC in HBV-infected patients has been practiced widely and been found to be useful in detecting HCC at early stage [2, 3]. However, the current HCC screening is reported as borderline cost effective in the Asia-Pacific region, and the optimal screening program remains to be established [4, 5]. One of the controversial issues is that when to begin this periodic screening in HBV infectors due to a wide variety of onset age of HCC. Knowledge on the factors that influence the onset age of HCC will improve current HCC surveillance program in HBV-infected patients.

Accumulating evidence from the last decade suggests that natural killer (NK) cells play an important role not only in controlling viral hepatitis but also in contributing to the pathogenesis of liver injury and inflammation [6]. For example, NK cell activation has been reported to play a critical role in liver inflammation during chronic HBV infection both in HBV transgenic mice and in HBV-infected patients [7–9]. Since persistent inflammation has been recognized as a driving force in HCC genesis [10, 11], NK cell activation may also be involved in HBV-related HCC development.

Killer cell immunoglobulin-like receptors *(KIR)* are involved in the regulation of NK cell activation through recognition of their human leukocyte antigen *(HLA)* class I ligands. This family of receptors consists of both activating and inhibitory allotypes. *KIRs* function can be predicted from the length of the cytoplasmic domain, where long receptors (*KIR2DL/KIR3DL*) are generally inhibitory and all short receptors (*KIR2DS/KIR3DS*) are activating. For inhibitory *KIRs,* the major ligands are *HLA-C* molecules. In terms of *KIR* recognition, all the allelic variants of *HLA-C* can be divided into two groups on the basis of alternative amino acids at position 80 of the extracellular domain. *HLA-C* group 1 alleles (*HLA-C1*) have an asparagine at position 80, whereas *HLA-C* group 2 alleles (*HLA-C2*) have a lysine at this position. *KIR2DL1* recognizes *HLA-C2* molecules, whereas *KIR2DL2* and *KIR2DL3* prefer *HLA-C1* molecules [12]. Some *HLA-B* and *HLA-A* alleles containing a homologous motif termed *Bw4* are known to bind to *KIR3DL1 *[13, 14]. Besides, some *HLA* class I molecules are reported as putative ligands (*HLA-C1-KIR2DS2*, *HLA-C2-KIR2DS1*, *HLA-Bw4-KIR3DS1,* and *HLA-A3/A11-KIR3DL2*) [15–18].

It has been clearly demonstrated that the strength of *HLA-KIR* interactions has functional significance and can influence disease susceptibility [19]. We found that the polymorphisms of *KIR* and *HLA* class I loci were associated with HCC occurrence in HBV-infected patients in a case-control study [20, 21]. We wondered whether the *KIR* and *HLA* genetic background could also influence the onset age of HBV-related HCC. HCC is a disease with a strong male dominance, and male patients usually have earlier onset age compared to female patients [1]. In this study, we focused on males and investigated the influence of *KIR* and *HLA* genetic background on the onset age of HBV-related HCC.

## 2. Methods

### 2.1. Patients

One hundred and seventy-one unrelated patients were enrolled from the Second Affiliated Hospital of Southeast University. Among them, 114 patients were enrolled from 2005 to 2007, and 57 patients were enrolled from 2011 to 2012. The patients were selected according to the following criteria: (a) being diagnosed as having primary HCC; (b) being males; (c) hepatitis B surface antigen (HBsAg) being positive for more than six months; (d) having hepatic ultrasonography within the past 12 months before they were diagnosed as having HCC; and (e) being members of Han population and living in the same geographical area. The exclusion criteria include: (a) being positive for other hepatitis viruses serum markers (hepatitis A virus IgM, hepatitis C virus antibody, hepatitis D virus antigen, hepatitis D virus antibody, and hepatitis E virus IgM) and human immunodeficiency virus antibody; (b) having an indication of autoimmune disease. All diagnoses of HCC were defined by clinical and biological criteria and confirmed by imaging technologies (defined as 1 or more tumoral nodules by computed tomography and ultrasonography). Eighty-six patients were also included in our previous case-control study [19]. The protocol was approved by the ethics committee of the Second Affiliated Hospital of Southeast University, and all the patients provided written, informed consent before enrollment.

### 2.2. Extraction of Genomic DNA

Genomic DNA was extracted from peripheral blood mononuclear cells by a standard salting-out method.

### 2.3. *KIR* Genotyping

PCR amplification with the primers specific for each locus of the following inhibitory *KIR* genes: *2DL1, 2DL2, 2DL3, 2DL5*, and *3DL1* and activating KIR genes: *2DS1, 2DS2, 2DS3, 2DS4(f) *(the full-length form of *2DS4*), *2DS4(d) *(the 22 bp-deletion mutant form of *2DS4*), *2DS5*, and* 3DS1* was performed as described in previous reports [20, 22]. The internal positive control primers for the fragment of the framework gene *KIR2DL4* were included in each PCR reaction. All primer sequences and amplification conditions are available upon request.

### 2.4. *HLA* Genotyping

*HLA-A, -B*, and -*C* were genotyped with high resolution by a routine sequence-based typing method [23]. Exons 2 and 3 of *HLA-A, HLA-B,* and *HLA-C* loci were amplified from genomic DNA by PCR using the locus-specific primers as described [23]. Sequencing reactions were performed using the BigDye Terminator v3.1 Cycle Sequencing Ready Reaction Kit (Applied Biosystems). Exons 2 and 3 of each locus were sequenced in both forward and reverse directions using a 3730XL DNA Analyzer (Applied Biosystems, Foster City, CA). The sequences were then analyzed using online dbMHC SBT typing tool [24]. One novel *HLA* class I allele was identified in this population. Nucleotide sequence of new allele has been submitted to the GenBank nucleotide sequence database and is available under the accession number EF468681 [25].

### 2.5. Statistical Methods

The effects of each *KIR* locus, *HLA* ligand, and* HLA-KIR* combination were examined individually by Kaplan-Meier (KM) analysis. *P* values were determined using Log Rank test for KM analysis. Univariate Cox proportional hazard regression model was applied to each demographic and clinical variable individually (see Table S3 in Supplementary Material available online at http://dx.doi.org/10.1155/2013/874514). After that, multivariate Cox regression analysis was employed to the genetic factors with *P* value less than 0.05 and the clinical variables with *P* value less than 0.5 (Table 4). *P* values generated through Cox modeling were supported with hazard ratios (HR) and 95% confidence intervals (CI). *HLA-C* genotype frequencies were checked for Hardy-Weinberg equilibrium using Pearson's *χ* ^2^ test. All the median ages were estimated by KM analysis. The final Cox regression model (Table 4) included 161 cases because the genotyping of some loci could not be accomplished for some cases due to lack of DNA. All statistical analyses were performed using SPSS software (Version 11.0 Chicago IL, SPSS Inc.).

## 3. Results

### 3.1. Patients' Demographic and Clinical Characteristics (Table 1)

All HCC patients selected were males with history of chronic hepatitis B, free of other hepatic virus coinfection, and with no indication of autoimmune disease. One hundred and forty-two patients (83.0%) were diagnosed as having cirrhosis, which was consistent with previous reports [26]. Sixty-three patients (36.8%) were at TNM stage III or IV when they were diagnosed as HCC. The HBeAg status and HBV DNA level presented in Table 1 were tested when the patients were diagnosed as having HCC. However, only 70 patients were quantified for HBV DNA in sera when they were diagnosed as having HCC. The onset age value was determined from KM survival-time data, with the 25th, 50th, and 75th percentiles of cancer free survival time being reported. The median onset age, defined as the age at which 50% of the population is cancer free, was 50 years. The distribution of onset ages of HCC is comparable with the recent report in Shanghai population [27].

### 3.2. Association of the Onset Age with *KIR* Polymorphisms (Table 2)

Twelve *KIR* genes were detected in this population. The result showed that *KIR2DL1* (97.7%), *KIR2DL3* (97.7%), and *KIR3DL1* (98.2%) were present in nearly all individuals. The frequencies of other *KIR* loci varied from 15.2% to 83.6%. KM analyses were performed on each of the *KIR* genes individually. No statistical significant result was found on any given *KIR* locus (Table 2).

### 3.3. Association of the Onset Age with *HLA* Ligands**  **and *HLA*-*KIR* Combinations

One hundred and forty-nine *HLA *class I alleles (37 *HLA-A* alleles, 60 *HLA-B* alleles, and 52 *HLA-C* alleles) were identified in these patients. To explore whether *HLA* ligands for *KIR* could influence the onset age of HBV-related HCC, we grouped *HLA* alleles according to *KIR* ligand as *Bw4, Bw4T, Bw4I, HLA-C1,* and *HLA-C2*. KM analyses were performed on *Bw4, Bw4T, Bw4I, HLA-C1C1, HLA-C1C2*, and* HLA-C2C2* individually. The frequencies of *HLA-C *genotypes were consistent with Hardy-Weinberg equilibrium (*P* = 0.44). Putative ligands of *HLA-A3 *and* -A11* were also analyzed. However, no significant result was found (Table S1). Then, we tested the effects of *Bw4-KIR* ligand-receptor (or putative ligand-receptor) combinations, including *Bw4-KIR3DL1, Bw4T-KIR3DL1, Bw4I-KIR3DL1, Bw4-KIR3DS1, Bw4T-KIR3DS1,* and *Bw4I-KIR3DS1*, on the onset age of HCC by KM analysis individually. No significant result was found (Table S2).

The effect of each *HLA-C-KIR* ligand-receptor (or putative ligand-receptor) combination on the onset age of HCC was then analyzed. There was high linkage disequilibrium in *KIR2DS2* and *KIR2DL2 *in our patients as in all populations tested to date (http://www.allelefrequencies.net/). Because our data did not allow to distinguish the effects between *C1C1-KIR2DS2* and *C1C1-KIR2DL2, *we combined *C1C1-KIR2DS2* and *C1C1-KIR2DL2* as *HLA-C1C1 *+ *KIR2DS2* or/and *KIR2DL2* (*C1C1-KIR2DS2/2DL2*). *C1C1-KIR2DS2/2DL2* was found to be associated with earlier onset age of HCC (the median age for the *C1C1-KIR2DS2/2DL2 *positive patients was 44 years, compared to 50 years for the *C1C1-KIR2DS2/2DL2 *negative patients, *P* = 0.004). No statistical significance was found on the other *HLA-C-KIR* ligand-receptor combinations (Table 3).

The effects of each demographic and clinical variable (including cirrhosis status, HBeAg status, TNM stage, family history of HBV-related diseases, and alcohol consumption) on the onset age of HCC were tested by univariate Cox model analysis (Table S3). To maintain statistical power, the demographic and clinical variables with *P* value less than 0.5 were included as the covariables in the multivariate Cox hazard regression model to analyze the effect of *C1C1-KIR2DS2/2DL2*. The result showed that *C1C1-KIR2DS2/2DL2 *was an independent risk factor for earlier onset age of HCC (HR = 1.70, 95% CI = 1.01–2.85, *P* = 0.04, Table 4).

## 4. Discussion

HCC surveillance in patients with chronic HBV infection has been recommended by various regional liver societies. Hepatic ultrasonography and alpha fetoprotein test every 6 months are the preferred program in China [28]. Although this periodic screening has been practiced widely, its benefits remain uncertain. Therefore, an optimal HCC surveillance program with acceptable cost effectiveness is needed, particularly for at-risk populations [4, 29]. One of the controversial issues is that when to begin this periodic screening in HBV-infected patients. The American Association for Study of Liver Disease recommends that, for noncirrhotic patients with hepatitis B, males above the age of 40 years and females above the age of 50 years are appropriate candidates for HCC surveillance [30]. The Asia-Pacific Association for Study of Liver Disease recommends the HCC surveillance program for high-risk patients with chronic hepatitis B (especially those who aged >30 years with serum HBV DNA levels >20 000 IU/mL) in the absence of a known diagnosis of cirrhosis [5]. The Ministry of Health of the People's Republic of China recommends lately that, for HBV-infected patients, males above the age of 40 years and females above the age of 50 years are appropriate candidates for HCC surveillance [28]. Knowledge on the factors that influence the age of HCC onset will provide basis for the improvement of the current HCC surveillance programs. HCC occurs mainly in men. Male : female ratios between 3 : 1 and 4 : 1 are reported in East China [1, 20, 27]. In this study, we examined the *KIR* and *HLA* genetic background in 171 male patients. We found that the median onset age of HCC was 6 years earlier in patients with a particular *HLA-KIR* combination of *C1C1-KIR2DS2/2DL2* than that of the patients without this combination (Table 4). The patients with *C1C1-KIR2DS2/2DL2* accounted for about 12% of this study cohort. Because both *KIR *genes and *HLA-C1* can be identified by real-time polymerase chain reaction [31], which is economical and time saving, the detection of *KIR2DS2/2DL2* and *HLA-C1 is* easy to be applied in clinical practice. Therefore, this study could help focus early-detection programs to a two-tiered model for greatest cost-benefit ratio.

In our previous case-control study, several *KIR* and *HLA* variants, including *HLA-C1C1*, *HLA-Bw4-80I, *and *KIR2DS4(f)/(d)*, were identified as the risk factors for HCC development in the patients with HBV infection. Because all of these risk factors we found had been reported to result in high NK cell functional potential, the data strongly suggested that overactivation of NK cell contributed to HBV-related HCC development [20]. The results of current study support the importance of NK cells (or other KIR expressing cells) in the progress of HBV-related HCC development. We could not distinguish the effect between *C1C1-2DS2* and *C1C1-2DL2 *because *KIR2DS2* and *KIR2DL2* were in high linkage disequilibrium. Since *KIR2DS2* delivers activating signal while *KIR2DL2 *delivers inhibiting signal, the correlation itself does not indicate the underlying mechanism. However, since there is little biologic difference between *KIR2DL2* and *KIR2DL3* (*KIR2DL2* and *KIR2DL3* are alleles of the same locus with the same *HLA* ligand) and *C1C1-2DL3 *has no effect on onset age of HCC (*P* = 0.81, Table 3), it seems more plausible that the activating* KIR2DS2* is the biologically relevant predictor.

According to the full combinations of *HLA-C* genotype and *KIR2DS2/2DL2*, the population can be divided into four groups:(a) *C1C1-2DS2/2DL2*, (b) *C1C1-2DS2/2DL2-*, (c) *C2-2DS2/2DL2*, and (d) *C2-2DS2/2DL2-*. Besides *C1C1-2DS2/2DL2*, the effects of other three groups on the onset age were also analyzed. The median onset ages for groups *C1C1-2DS2/2DL2-, C2-2DS2/2DL2, *and *C2-2DS2/2DL2-* were 49 years, 53 years, and 49 years, respectively. None of the combinations influenced the onset age of HCC significantly (*P* = 0.75, 0.28, and 0.31 by KM analyses, resp.). The median onset age of *C2-2DS2/2DL2* group was 9 years older than that of *C1C1-2DS2/2DL2* group (53 years versus 44 years, *P* = 0.03 by KM analysis). This result supports further that *KIR* and *HLA* combinations can influence the onset age of HCC.

To further refine programs for those at highest risk of unresectable HCC, we explored the association between *KIR* locus, *HLA* ligand, and *HLA-KIR* combination with the TNM stages of HCC at diagnosis. However, no statistical significance was found (data not shown).

The determination of HCC onset is rather difficult because most HCC patients are asymptomatic at the early stage of HCC. In general, many HBV-infected patients take regular abdomen ultrasonography in a biannual HCC surveillance program or in an annual routine medical examination. To determine the precise onset of HCC, we selected patients who had taken abdomen ultrasonography within the past 12 months before they were diagnosed as HCC, which means that these patients had not developed HCC (or ultrasonography-detectable HCC) one year before their diagnosis. Therefore, the onset of HCC was defined as the time of first diagnosis in this study.

Virological factors, such as HBeAg status, HBV viral load, HBV genotype, and HBV mutations, are reported to influence the HCC risk, although few reports concern the onset age of HCC [32]. Population-based cohort studies support that positive for HBeAg associated with increased HCC risk [33, 34]. Our results showed that positive for HBeAg at diagnosis had no influence on the onset age of HCC (HR = 1.17, *P* = 0.38, Table S3). Level of HBV viraemia from the age of 30 years was reported as an independent risk factor for HCC during the next decade [33]. However, the result of univariate Cox analysis showed that the HBV viral load, at their diagnosis as HCC, had no influence on the onset age of HCC in our study population (HR = 1.00, *P* = 0.89, Table S3). HBV genotype C in Asian cohorts [35], genotype F in other populations [36], and some HBV mutations [37] were reported to increase the risk of HCC. One limitation of our study is lack of information of HBV genotype and mutations in the patients.

## 5. Conclusion

**Figure 1:**
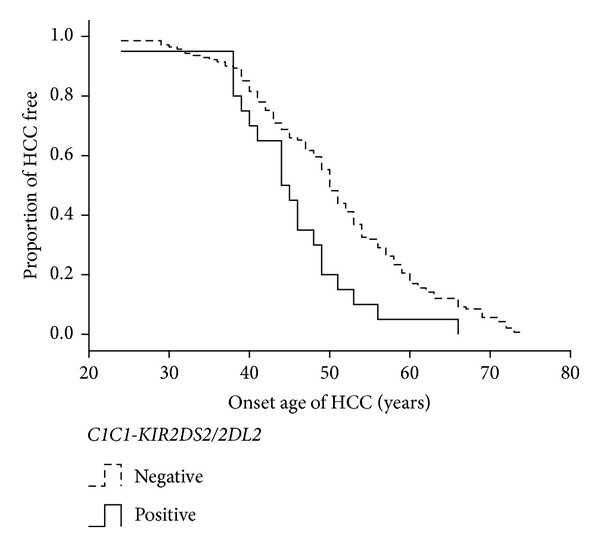
The effect of *C1C1-KIR2DS2/2DL2* on the onset age of HCC by KM survival analysis. The plot shows the effect of *C1C1-KIR2DS2/2DL2* on the onset age of HCC in males with HBV infection (*n* = 161). *C1C1-KIR2DS2/2DL2* positive patients have earlier onset age of HCC (*P* = 0.004).

The present study shows that *C1C1-2DS2/2DL2* is an independent risk factor for earlier onset age of HCC (Figure 1, Table 4) in male patients with HBV infection. To our knowledge, this is the first report on the association between *KIR* and *HLA* genetic background and the onset age of HCC. Our findings may be useful to improve the current HCC surveillance program in local HBV-infected patients. Additionally, this observation along with our previous case-control study suggests an important role of NK cell (or other *KIR*-expressing cell) in the process of HCC development during HBV infection. Future studies on the mechanism underlying these genetic associations may provide insights for new therapeutic strategies in preventing HCC in HBV-infected patients.

## Supplementary Material

There are three tables in Supplementary Material. Table S1 describes the effects of HLA as KIR ligand on the onset age of HCC by KM analysis. Table S2 describes the effects of Bw4-KIR ligand-receptor combinations on the onset age of HCC by KM analysis. While Table S3 describes the univariate Cox model survival analyses of demographic and clinical characteristics of 171 male HCC patients.

## Figures and Tables

**Table 1:** Demographic and clinical characteristics of 171 male HCC patients.

	Number of cases	Percentage
Age of onset^†^		
Median (IQR)^‡^	50 (42~57)	—
Range	24~74	—
Cirrhosis	142	83.0.%
TNM stage III or IV	63	36.8%
HBsAg positive	171	100%
HBeAg positive	60	35.1%
HBV DNA copy number^†§^		
Mean	2.3*E* + 06	—
Range	0~4.8*E* + 07	—
Family history of HCC	9	5.3%
Family history of HBV-related diseases^¶^	43	25.1%
Alcohol consumption (≥1 drink per week)	20	11.7%

^†^Age of first diagnosis for HCC.

^‡^Age is presented as median, interquartile range (IQR), which are determined by KM estimates.

^§^70 patients were quantified for HBV DNA when they were diagnosed as HCC.

^¶^HBV-related diseases include HBV-related hepatitis, cirrhosis, liver failure, and HCC.

**Table 2:** Effects of *KIR* genes and genotypes on the onset age of HCC by KM analysis.

*KIR* gene and genotype		*n* (%)	Median age	*P* value
*2DL1 *	Negative	4 (2.3)	45	0.82
	Positive	167 (97.7)	50	
*2DL2 *	Negative	137 (80.1)	50	0.09
	Positive	34 (19.9))	49	
*2DL3 *	Negative	4 (2.3)	45	0.81
	Positive	167 (97.7))	50	
*2DL5 *	Negative	103 (60.2)	50	0.67
	Positive	68 (39.8)	50	
*3DL1 *	Negative	3 (1.8)	41	0.68
	Positive	168 (98.2)	50	
*2DS1 *	Negative	109 (63.7)	50	0.49
	Positive	62 (36.3)	49	
*2DS2 *	Negative	139 (81.3)	50	0.09
	Positive	32 (18.7)	50	
*2DS3 *	Negative	145 (84.8)	50	0.07
	Positive	26 (15.2)	49	
*2DS4(f) *	Negative	28 (16.4)	49	0.83
	Positive	143 (83.6)	50	
*2DS4(d) *	Negative	116 (67.8)	50	0.97
	Positive	55 (32.2)	49	
*2DS5 *	Negative	124 (72.5)	50	0.50
	Positive	47 (27.5)	51	
*3DS1 *	Negative	108 (63.2)	49	0.98
	Positive	63 (36.8)	50	
*KIR* genotype	AA	84 (49.1)	50	0.42
	BX	87 (50.9)	50	

**Table 3:** Effects of *HLA-C-KIR* ligand-receptor combinations on the onset age of HCC by KM analysis.

*HLA-KIR* combination		*n*	Median age	*P* value
*C1C1-KIR2DL3 *	Negative	40	50	0.81
	Positive	98	47	
*C1C2-KIR2DL3 *	Negative	109	48	0.78
	Positive	29	50	
*C1C2-KIR2DL1 *	Negative	110	49	0.65
	Positive	28	50	
*C2C2-KIR2DL1 *	Negative	131	49	0.81
	Positive	7	50	
*C1C1-KIR2DS2/2DL2 *	Negative	141	50	0.004**
	Positive	20	44	
*C1C2-KIR2DS2/2DL2 *	Negative	155	49	0.53
	Positive	8	53	
*C1C2-KIR2DS1 *	Negative	134	50	0.68
	Positive	7	50	
*C2C2-KIR2DS1 *	Negative	138	50	0.12
	Positive	3	43	

**P < 0.01.

**Table 4:** Multivariate Cox model survival analysis of *C1C1+2DS2/2DL2* and other factors on the onset age of HCC.

Factor	*P* value	HR	95% CI
*C1C1+2DS2/2DL2 *	0.04*	1.70	1.01–2.85
Cirrhosis	0.03	0.59	0.36–0.96
HBeAg positive	0.39	1.19	0.80–1.77
Family history of HBV-related diseases	0.23	1.30	0.85–2.01

*P < 0.05.
