# Supplementary material for: Combination of Human Leukocyte Antigen and Killer Cell Immunoglobulin-Like Receptor Genetic Background Influences the Onset Age of Hepatocellular Carcinoma in Male Patients with Hepatitis B Virus Infection
Source: Clin Dev Immunol. 2013 Nov 10;2013:874514. doi: 10.1155/2013/874514 (PMC3842051; doi:10.1155/2013/874514)
Supplement: Supplementary file 1 — There are three tables in Supplementary Material. Table S1 describes the effects of HLA as KIR ligand on the onset age of HCC by KM analysis. Table S2 describes the effects of Bw4-KIR ligand-receptor combinations on the onset age of HCC by KM analysis. While Table S3 describes the univariate Cox model survival analyses of demographic and clinical characteristics of 171 male HCC patients. [file 874514.f1.doc]

Table S1 Effects of *HLA* as *KIR* ligand on the onset age of HCC by KM analysis

| *KIR* gene |  | n | Median age | P value |
| --- | --- | --- | --- | --- |
| *Bw4* | negative | 71 | 49 | 0.82 |
|  | positive | 85 | 50 |  |
| *Bw4T* | negative | 102 | 50 | 0.64 |
|  | positive | 54 | 49 |  |
| *Bw4I* | negative | 107 | 49 | 0.57 |
|  | positive | 49 | 50 |  |
| *C1C1* | negative | 36 | 50 | 0.90 |
|  | positive | 102 | 48 |  |
| *C1C2* | negative | 109 | 48 | 0.78 |
|  | positive | 29 | 50 |  |
| *C2C2* | negative | 131 | 49 | 0.81 |
|  | positive | 7 | 50 |  |
| *A3* or *A11* | negative | 91 | 50 | 0.66 |
|  | positive | 65 | 50 |  |

Table S2 Effects of *Bw4-KIR* ligand-receptor combinations on the onset age of HCC by KM analysis

| *HLA -KIR* combination |  | n | Median age | P value |
| --- | --- | --- | --- | --- |
| *Bw4-KIR3DL1* | negative | 73 | 49 | 0.74 |
|  | positive | 83 | 50 |  |
| *Bw4T-KIR3DL1* | negative | 104 | 50 | 0.52 |
|  | positive | 52 | 49 |  |
| *Bw4I-KIR3DL1* | negative | 107 | 49 | 0.57 |
|  | positive | 49 | 50 |  |
| *Bw4-KIR3DS1* | negative | 138 | 50 | 0.70 |
|  | positive | 25 | 50 |  |
| *Bw4T-KIR3DS1* | negative | 145 | 50 | 0.27 |
|  | positive | 18 | 49 |  |
| *Bw4I-KIR3DS1* | negative | 148 | 49 | 0.97 |
|  | positive | 16 | 50 |  |

Table S3 Univariate Cox model survival analyses of demographic and clinical characteristics of 171 male HCC patients

| factor | P value | HR |
| --- | --- | --- |
| Cirrhosis | 0.04 | 0.63 |
| TNM stage III or IV | 0.89 | 1.03 |
| HBeAg positive | 0.38 | 1.17 |
| HBV DNA copy number† | 0.89 | 1.00 |
| Family history of HCC | 0.59 | 0.82 |
| Family history of HBV-related diseases | 0.27 | 1.22 |
| Alcohol consumption | 0.80 | 0.94 |

†, 70 patients were quantified for HBV DNA when they were diagnosed as HCC.
